# Establishment and evaluation of a CT-based radiomic model for AIDS-associated pulmonary cryptococcosis

**DOI:** 10.1186/s12880-022-00910-6

**Published:** 2022-10-29

**Authors:** Zi-xin Zhang, Xing-yu Mu, Jing Yu, Chun-shuang Guan, Bu-dong Chen, Ru-Ming Xie

**Affiliations:** 1 Department of Radiology, Beijing Ditan Hospital, Capital Medical University, Beijing, China; 2 Capital Medical University Electric Teaching Hospital, Beijing, China; 3 Beijing Yizhun Medical AI Technology Limited Company, Beijing, China

**Keywords:** Radiomic model, CT, AIDS, Pulmonary cryptococcosis

## Abstract

**Background:**

Establish a CT-based diagnostic radiomic model for AIDS complicated with pulmonary cryptococcosis and evaluate the diagnostic efficacy of this model.

**Methods:**

This retrospective study enrolled 98 AIDS patients with pulmonary cryptococcosis and 103 AIDS patients with other infections or neoplastic lesions, comprising a total of 699 lesions. Patients were randomly divided into a training group and test group at a ratio of 2.75:1. Features from all lesions, cavity lesions and solid nodule lesions were extracted, and two kinds of radiomic models (6 types) were established. ROC curves were drawn, and the sensitivity and specificity were calculated to compare the SVM model and LR model, radiologists’ empirical diagnoses and the combination of these empirical diagnoses with the radiomic model.

**Results:**

The AUCs of senior radiologist for all lesions and cavity lesions were lower than those of the SVM and LR models. The diagnostic efficacy of primary radiologist was lower than that of both of the other model types. The diagnostic efficacy of the LR model was relatively stable, with the highest diagnostic efficiency of the 3 model/radiologist groups. The AUCs of intermediate radiologist in combination with the LR radiomic model for all lesions, nodular lesions and cavity lesions were 0.88, 0.84, and 0.9, respectively, which were the highest among all models and radiologists.

**Conclusions:**

The CT-based radiomic LR model of AIDS-associated pulmonary cryptococcosis exhibits good diagnostic performance, which was similar to that of senior radiologists and higher than that of the primary radiologist. With the help of a radiomic model, radiologists can achieve improved diagnostic accuracy compared to that when only an empirical diagnosis is used.

**Supplementary Information:**

The online version contains supplementary material available at 10.1186/s12880-022-00910-6.

## Background

Cryptococcal pneumonia is an opportunistic infection with an incidence in the lungs second only to that of *Aspergillus* infection, and it remains the most fatal fungal disease among immunocompromised patients worldwide. It belongs to the category of invasive pulmonary fungal infections (IPFIs) and easily invades HIV-infected patients at a late stage during AIDS, causing high morbidity and mortality if not diagnosed and treated in time. Pulmonary cryptococcosis is transmitted through the respiratory tract, and fungal spores deposit in the alveoli to form colonies that spread to surrounding tissues or other organs when immunity is significantly reduced [1]. Both the symptoms and CT imaging features of cryptococcal pneumonia are not specific and need to be differentiated from other concomitant infectious or neoplastic diseases common in AIDS patients.

Radiomics, which is defined as the high-throughput automated (or semiautomated) extraction of large amounts of quantifiable information from a region of interest (ROI) on radiographic images, has aroused widespread interest. Radiomics is regarded as a class of machine learning algorithms that combine raw inputs into mid-layer features and has recently shown impressive results in many areas, especially in diagnostic radiology [2, 3]. At present, studies on radiomics in various human systems have been reported, but they mainly focus on patients with a normal immune system [4, 5], while there are few related studies on patients with immune deficiency [6], especially AIDS patients. AIDS patients are susceptible to opportunistic infections, and the incidence of some related malignant tumors is also higher than that of other patients. Early and accurate diagnosis can help improve the survival rate and prognosis of AIDS patients.

Therefore, the purpose of the present study was to establish a CT-based radiomic model for patients with AIDS complicated by cryptococcal pneumonia, analyze its diagnostic efficacy, and compare its diagnostic accuracy with that of radiologists with different levels of expertise to explore the application value of the model.

## Methods

### Patients

The study was approved by ethical committee of Beijing Ditan Hospital, Capital Medical University, written informed consent from the patients for use of data was waived by ethical committee of Beijing Ditan Hospital, Capital Medical University due to retrospective nature of the study. The following medical record data was collected from a single-center study carried out in Beijing Ditan Hospital from June 2016 to June 2021: age, sex, clinical features, CD4 cell count, CD8 cell count, chest CT scan imaging results, etc. During the study period, according to the HIV virus test, a total of 1490 AIDS patients were rechecked and confirmed, 208 patients without complete CT and clinical data, 366 patients had negative CT pulmonary imaging at the first visit, 85 patients had no nodules, cavitation or consolidation in the lung, 630 cases without histopathological or cytological diagnosis. Finally, A total of 201 HIV-infected patients were enrolled, among whom 98 patients were diagnosed with pulmonary cryptococcosis and 103 patients were diagnosed with noncryptococcal pneumonia, including 22 patients infected with *Aspergillus*, 13 with *Candida albicans*, 15 with *Penicillium marneffei*, 26 with pulmonary tuberculosis, 6 with NTM, 5 with Kaposi's sarcoma, 6 with pulmonary lymphoma and 10 with lung cancer. According to the radiological characteristics of the AIDS patients with pulmonary cryptococcosis [7–10], the lung lesions were mainly classified into three patterns based on the lesion morphology: solid nodules, cavitation, and consolidation. The inclusion criteria of the noncryptococcal pneumonia group were determined according to the above three morphological characteristics.

### CT image acquisition and data processing

Philips Discovery CT750HD and GE Light Speed VCT scanners were used to scan from the tip to the bottom of the lung. The scanning parameters were as follows: tube voltage, 120 kVp; automatic tube current, mAs; scanning thickness, 5 mm; image matrix, 512 × 512; reconstruction section thickness, 1.25 mm; reconstruction interval, 1.25 mm; and gantry rotation speed, 8 s. Coronal multiplanar reconstruction was also performed. For enhanced scanning, 80–100 ml of the nonionic contrast agent iodihydramol (300 mg/mL, 1.5 mL/kg) was injected through the elbow vein by the intravenous mass infusion method at a rate of 3 ml/s. Arterial and venous images were collected at 30 s and 60 s, respectively. Standard lung window imaging (width: 1600, window position: − 600) was selected, and images were saved in DICOM format. Through DcmAnonyRel-1 medical image anonymous software, personal information such as the patient’s name, test ID and test time was removed before uploading to the “Darwin Platform” launched by Beijing Yizhun AI Technology Co., Ltd. (http://211.145.67.46:9160/static/login_ct.html), which was used to extract and select radiomic features to build machine learning models.

### Lesion segmentation and radiomic feature extraction

**Fig. 1:**
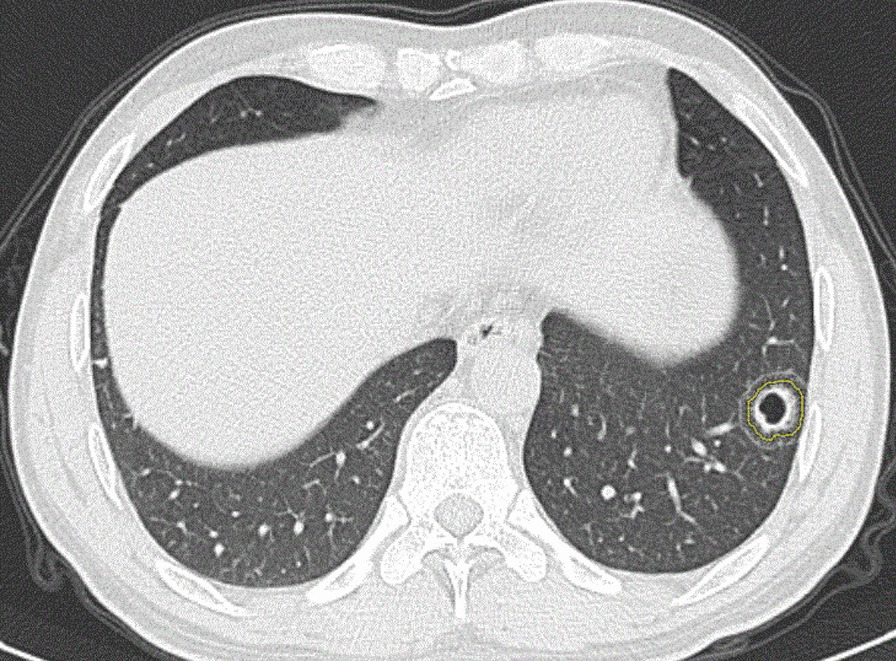
A 25-year-old male with AIDS complicated by Cryptococcus pneumonia presented to the hospital with fever for 2 weeks. A CT scan showed a thin-walled cavity in the lower lobe of the left lung. The automatic machine delineated the lesion approximately 1–2 mm from its outer edge

**Fig. 2:**
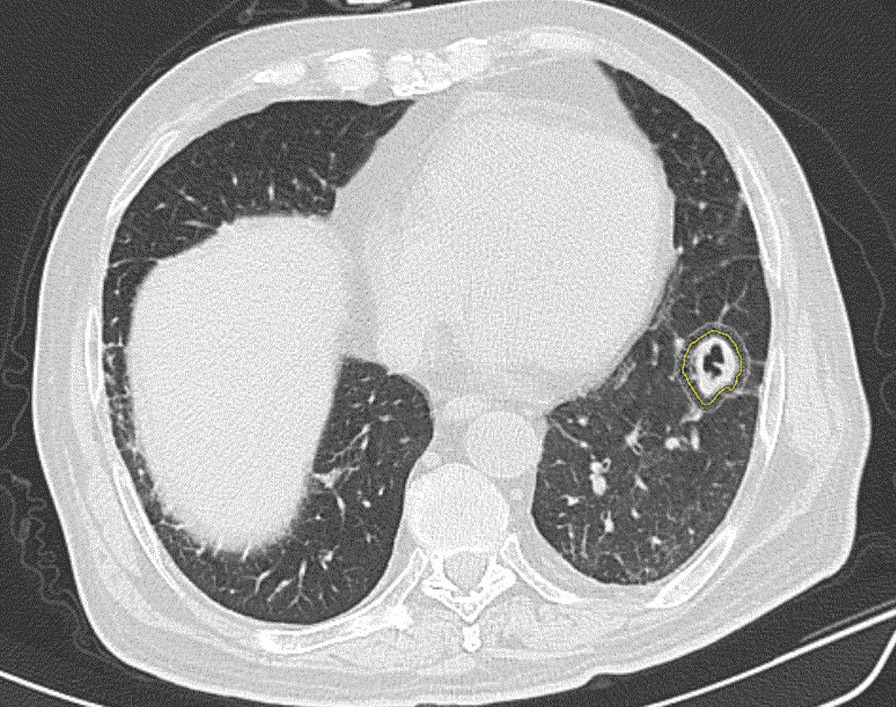
A 42-year-old man with AIDS and invasive aspergillosis presented with a 3-week history of expectoration. CT showed a thin-walled cavity lesion in the lower lobe of the left lung. The ground-glass opacity component was observed in the right outer edge of the lesion, and the radiologist manually adjusted the lesion edge after automatic machine delineation to include the GGO

Two radiologists in the diagnosis of pulmonary infectious diseases were selected to perform lesion segmentation according to the following rules: (1) Semiautomatic segmentation was used (machine with recognition of lesion location and rough delineation, manual adjustment of lesion boundary). (2) A nonenhanced image was used to delineate the standard lung window, excluding bronchus and pulmonary arteries/veins within the segmentation. (3) The dividing line of the region of interest (ROI) was approximately 1–2 mm from the edge of the lesion. (4) Lesion was segmented continuously layer by layer. (5) For single and multiple lesions (number < 5), each lesion was segmented, and diffuse lesions were segmented into at most 5 regions, as shown in Figs. 1 and 2. Radiologists-1(Senior radiologist: 18 years of experience in infectious disease diagnosis) review and revised the delineated lesion, radiologist-2(The Primary radiologist: 6 years of experience in infectious disease diagnosis) was responsible for the initial lesion contour segmentation. After segmentation, 3 categories of CT-based radiomic features were extracted: shape features, first-order features and texture features. Shape features and first-order features were derived from wavelet filter. Among the texture features that capture the spatial interdependence of voxels in images, 5 kinds of features were included: gray-level dependence matrix (GLDM), gray-level size zone matrix (GLSZM), gray-level cooccurrence matrices (GLCM), gray-level run-length matrices (GLRLM) and neighboring gray-tone difference matrix (NGTDM). Ultimately, the original image provided 136 quantitative features, and 1781 filter-based features were extracted from chest CT radiomics images. The extraction parameter details and recursive feature elimination (RFE) data are described in Additional file 1: 1.

### Radiomic feature selection and radiomic model establishment

**Fig. 3:**
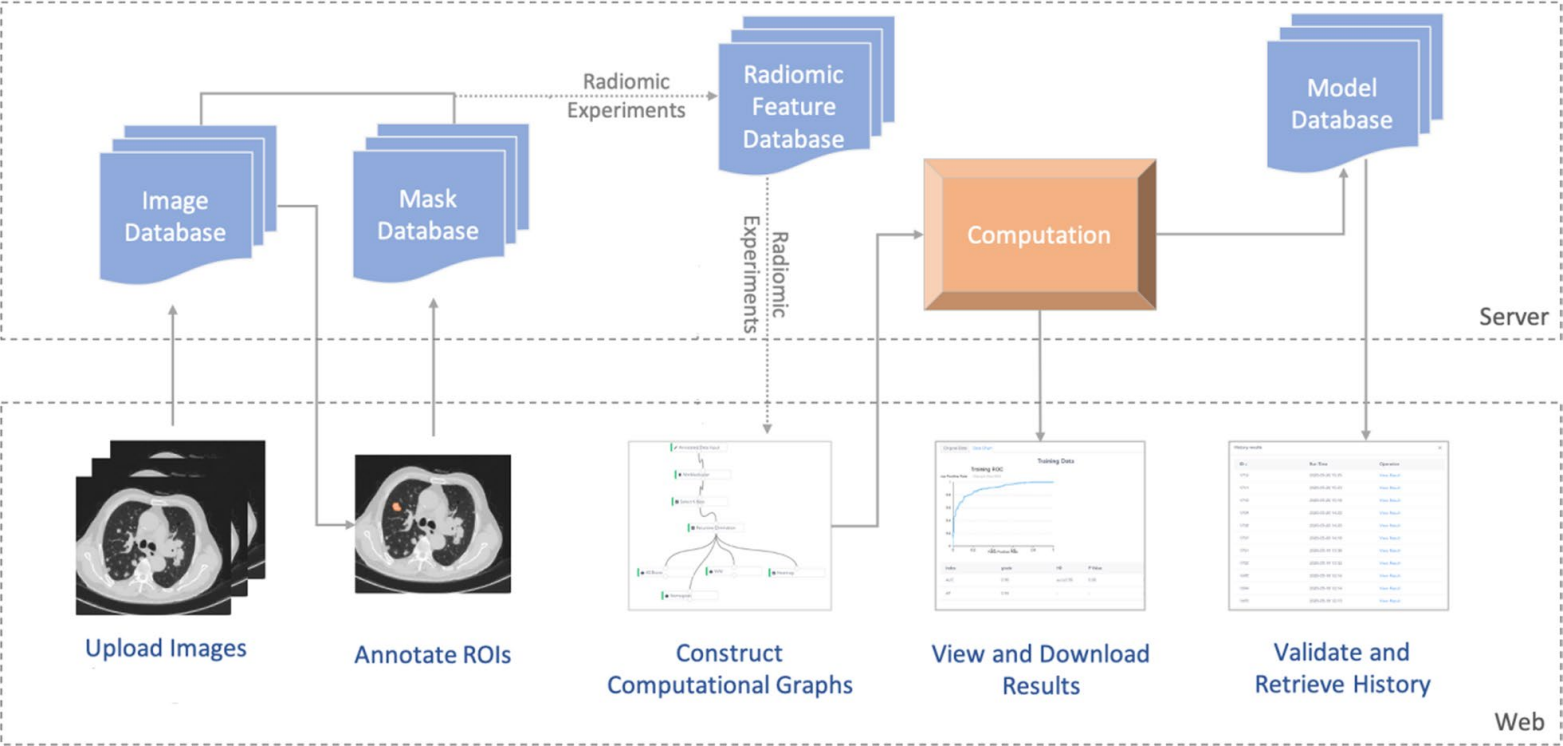
Flowchart for model establishment

The flowchart for model establishment is shown in Fig. 3. All patients were divided randomly into a training cohort and test cohort at a ratio of 2.75:1 by using stratified sampling. In order to avoid introducing bias, feature selection was applied only to the training group. All lesions, nodular lesions and cavity lesions were calculated separately. Before feature selection, the ‘standard scaler’ was selected as the preprocessing component to normalize the original data, making the algorithm converge rapidly and reasonably. To eliminate many redundant features in the original data, feature selection components were utilized. ‘Select K percentile’—‘recursive feature elimination’—‘select from model’ were carried out sequentially. In the processor component, ‘support vector machine’ (SVM) and ‘logistic regression’ (LR) were selected to build two different models for comparing the diagnostic performance. For the visualization component, a ‘heatmap’ was used to display the differences in feature distribution in different categories.

### Performance evaluation and statistical analysis

For the SVM and LR models, classification and calculation were performed according to the morphology of lesions, all lesions, nodular lesions and cavity lesions were trained and tested to obtain the diagnostic efficacy of each model, which was illustrated by receiver operating characteristic (ROC) curves. The area under the ROC curve (AUC) and the corresponding 95% confidence interval (95% CI) were calculated per lesion, as well as the sensitivity and specificity of each model. Additionally, radiologist-1, radiologist-2, radiologists-3(Intermediate radiologist:10 years of experience in infectious disease diagnosis) blinded to the pathological findings marked the location, border, density, and morphology of lung lesions as well as pleural effusion and mediastinal lymph nodes and made a diagnosis. According to the presence or absence of CT imaging signs and whether cryptococcal infection or not, the labels were marked, “1” for positive while “0” for negative status. After saving the data from the radiologists-3 as CSV format, the variables with a p < 0.1 on multivariate backward stepwise logistic regression with minimum akaike information criterion (AIC) was performed through the input channel of the “Darwin platform” to obtain features with differential diagnosis value. On the basis of multifactor logistic regression analysis, combined with the radiomics score of each patient, finally, the features of CT imaging as well as the established LR model were comprehensively calculated on the platform to obtain a final diagnosis. The performance was compared among the two models, radiologist-1 and radiologist-2’s empirical diagnoses and the combination of radiologists-3’s empirical diagnoses with the radiomic models.

All data were analyzed with SPSS 22.0 (IBM Corp.). The clinical indicators were expressed as the median value and interquartile range (IQR) as well as the mean ± standard deviation. Independent chi-squared tests and T tests were used for normally distributed data, and Kruskal–Wallis H tests were used for skewed data. The interobserver agreement regarding the 2 radiologists was calculated using the intraclass correlation coefficient (ICC). A p value < 0.05 was considered the standard for a statistically significant difference, and an ICC > 0.8 indicated good agreement.

## Results

### Basic clinical characteristics and radiological features

The clinical characteristics and CT characteristics are shown in Table 1. Among all enrolled HIV-infected patients, the proportion of male patients was significantly higher than that of female patients. Cell counts were significantly below the normal range, indicating impaired immunity, which was not different among groups. Fever was the main presentation of patients at the time of visit, accounting for the highest proportion of patients in each group, but there was no statistically significant difference between various clinical symptoms. Among basic CT characteristics, the number and location of lesions, lymph nodes, pleural effusion and other influencing manifestations were recorded for analysis. There were more enlarged lymph nodes in the noncryptococcal pneumonia group than in the pulmonary cryptococcosis group (p = 0.001). The probability of pleural effusion in the testing group was significantly lower than that in the training group (p = 0.008).

**Table 1:** Clinical and radiological characteristics

Characteristics N(%)		Cryptococcosis	Non-cryptococcosis	*p* value	Training group	Testing group	*p* value
Sex	Male	89 (90.82)	90 (85.71)	0.502	132 (89.19)	47 (88.68)	0.549
	Female	9 (9.18)	13 (12.38)		16 (10.81)	6 (11.32)	
Age (years)	Mean ± SD	37.1 ± 11.2	42.0 ± 14.5	0.09	39.6 ± 13.5	39.8 ± 12.6	0.928
	Range	19–77	20–92		19–92	22–77	
Cell count (cells/μl) (p50,IQR)	CD4	31.5 (14, 73.5)	44 (14, 104)	0.232	39 (14, 97.5)	34 (14, 75.5)	0.369
	CD8	461.5 (240.5, 872)	378 (222, 694)	0.109	439 (226.3, 845.7)	341 (229, 638)	0.230
	CD4/8 ratio	0.65 (0.375, 0.143)	0.13 (0.06,0.21)	**0.01**	0.08(0.43, 0.18)	0.09 (0.05, 0.20)	0.703
Symptom	Fever	63 (37.06)	70 (38.89)	0.402	93 (37.05)	39 (22.29)	0.985
	Respiratory symptoms	46 (27.06)	57 (31.67)		76 (30.28)	32 (18.29)	
	Other symptoms	61 (35.88)	53 (29.44)		82 (32.67)	104 (59.43)	
Lesions count	Single	42 (42.86)	48 (45.71)	0.671	67 (45.27)	23 (43.40)	0.873
	Multiple	56 (57.14)	55 (52.38)		81 (54.73)	30 (56.60)	
Location	Right Lung	31 (31.63)	24 (22.86)	0.153	39 (26.35)	16 (30.19)	0.459
	Left lung	38 (38.78)	37 (35.24)		54 (36.49)	21 (39.62)	
	Bilateral Lung	29 (29.59)	44 (41.90)		58 (39.19)	15 (28.30)	
Other features	Enlarged lymph node	24 (24.49)	49 (46.67)	**0.001**	55 (37.16)	18 (33.96)	0.621
	Pleural effusion	34 (34.69)	31 (29.52)	0.430	56 (37.83)	9 (16.98)	**0.008**

Bold was considered a statistically significant difference

### CT-based radiomic models and performance evaluation

**Fig. 4:**
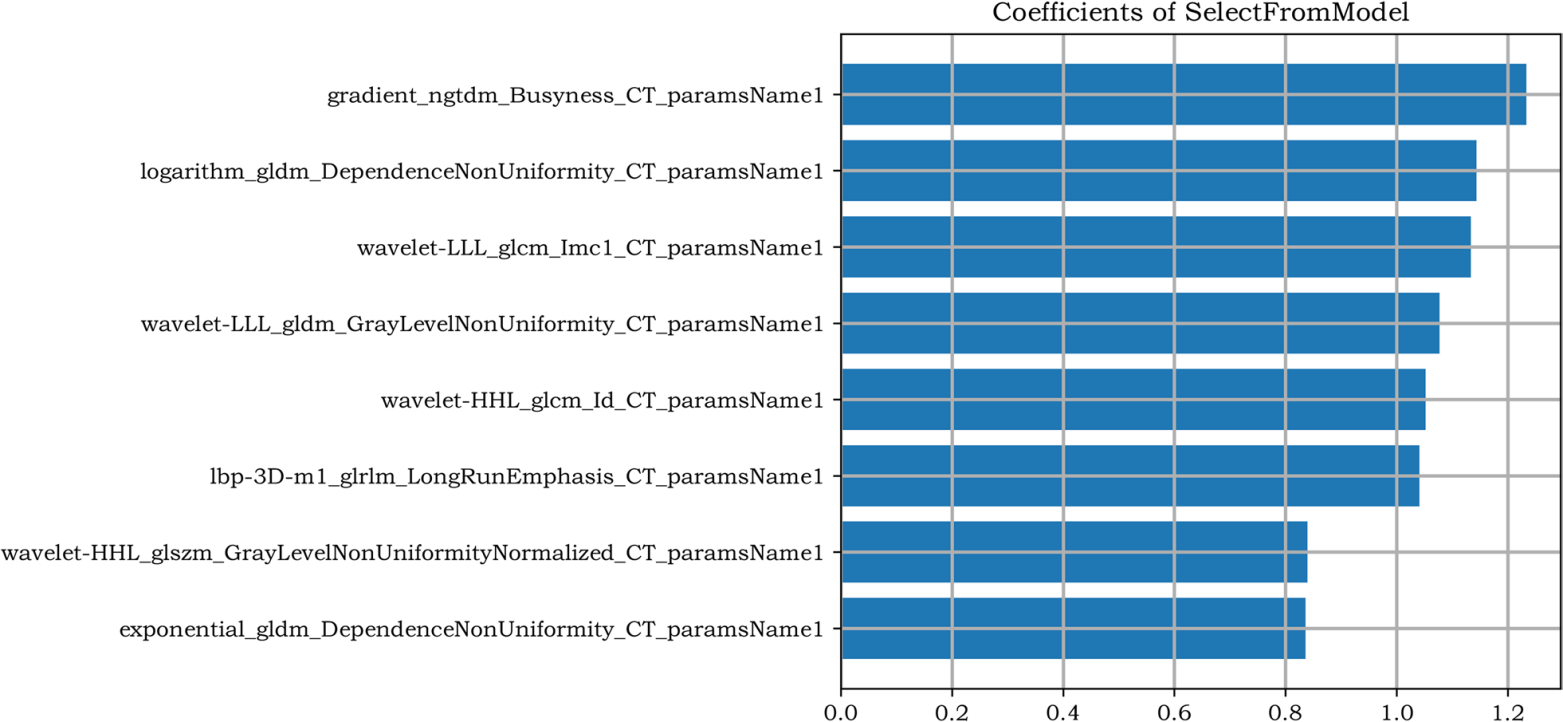
The 8 most valuable features were screened out during preprocessing and feature screening

**Fig. 5:**
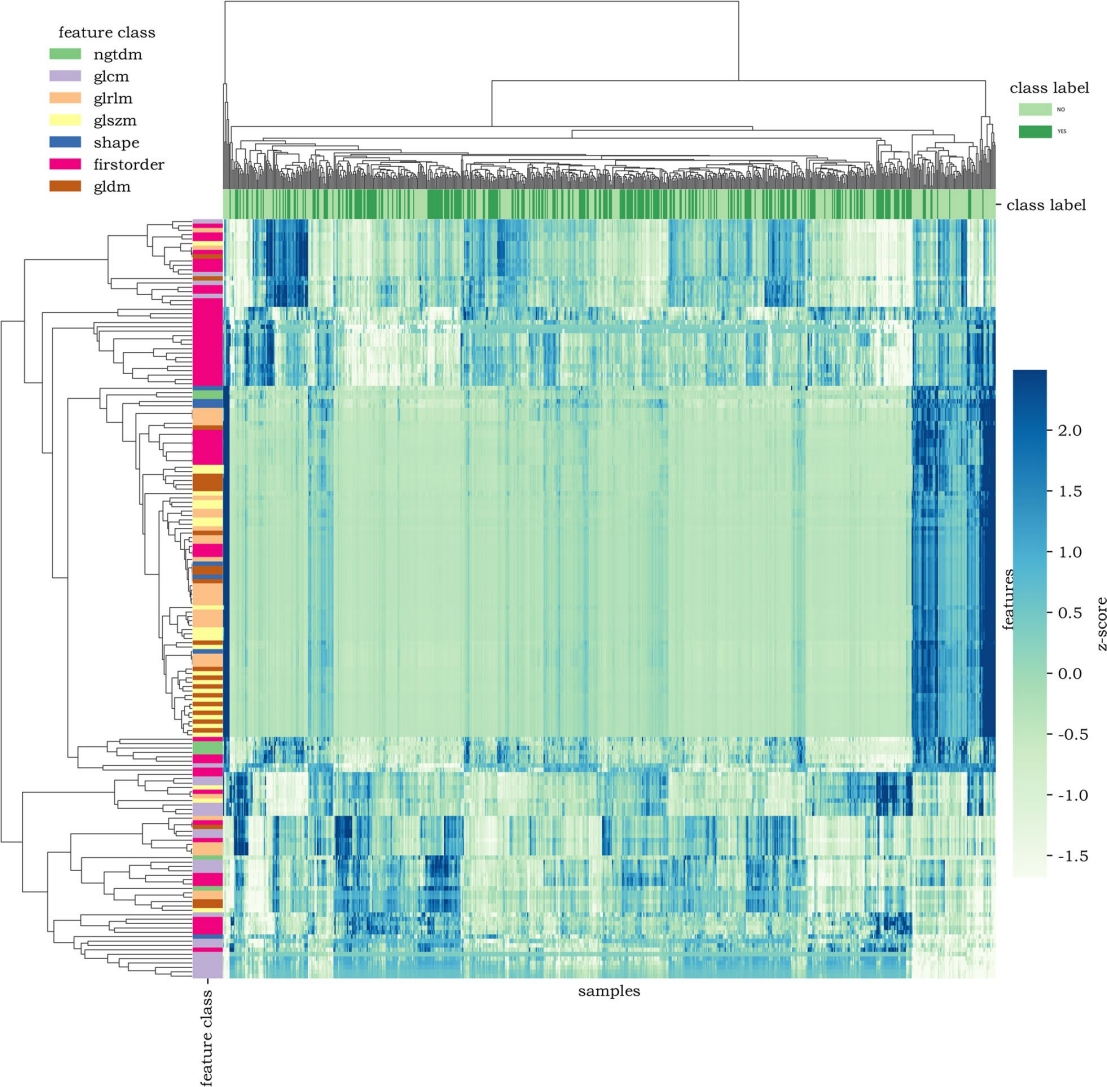
The ‘heatmap’ of the selected radiomics features

**Fig. 6:**
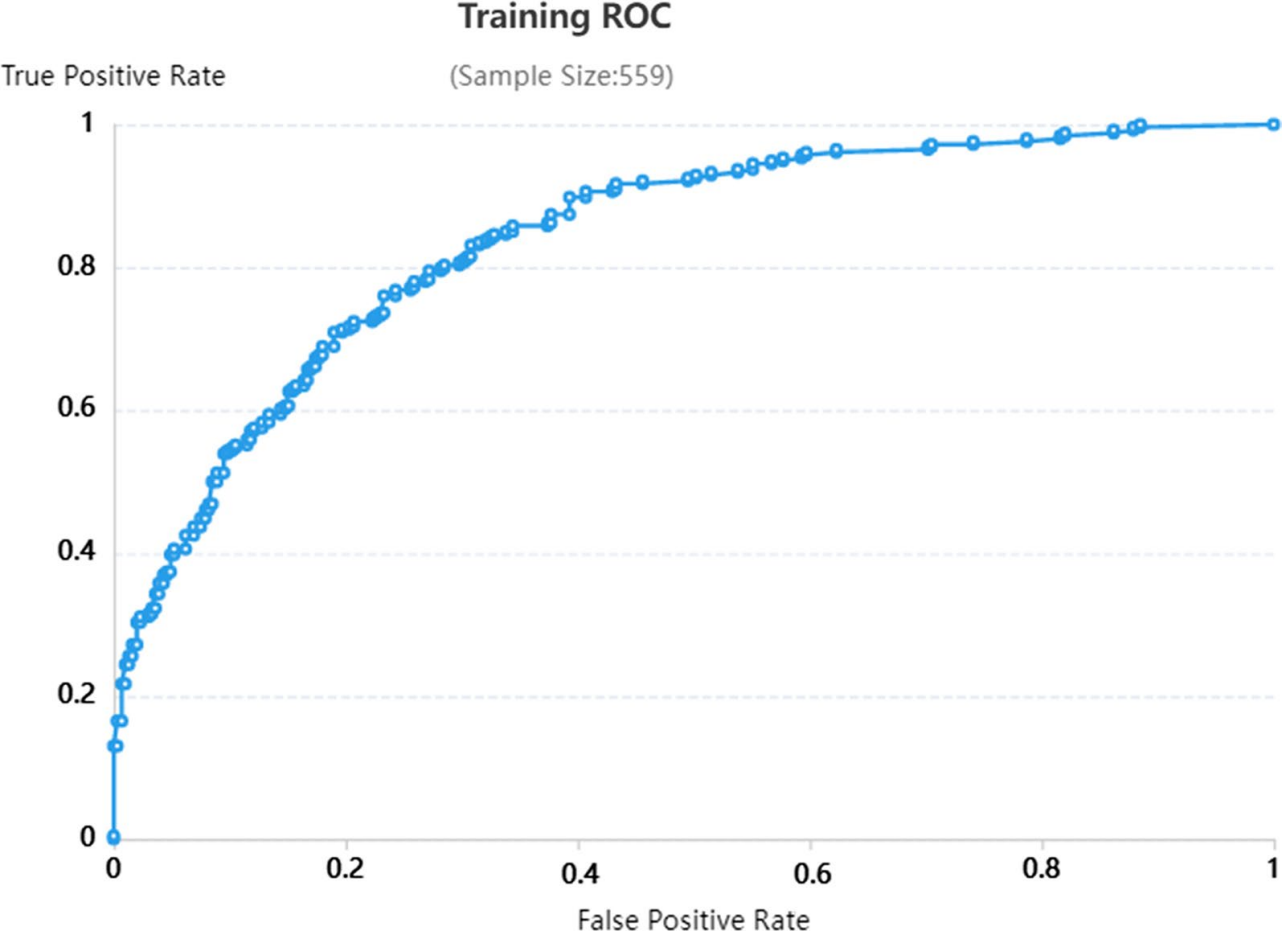
ROC curves for all lesions in the training group with the LR radiomics model

**Fig. 7:**
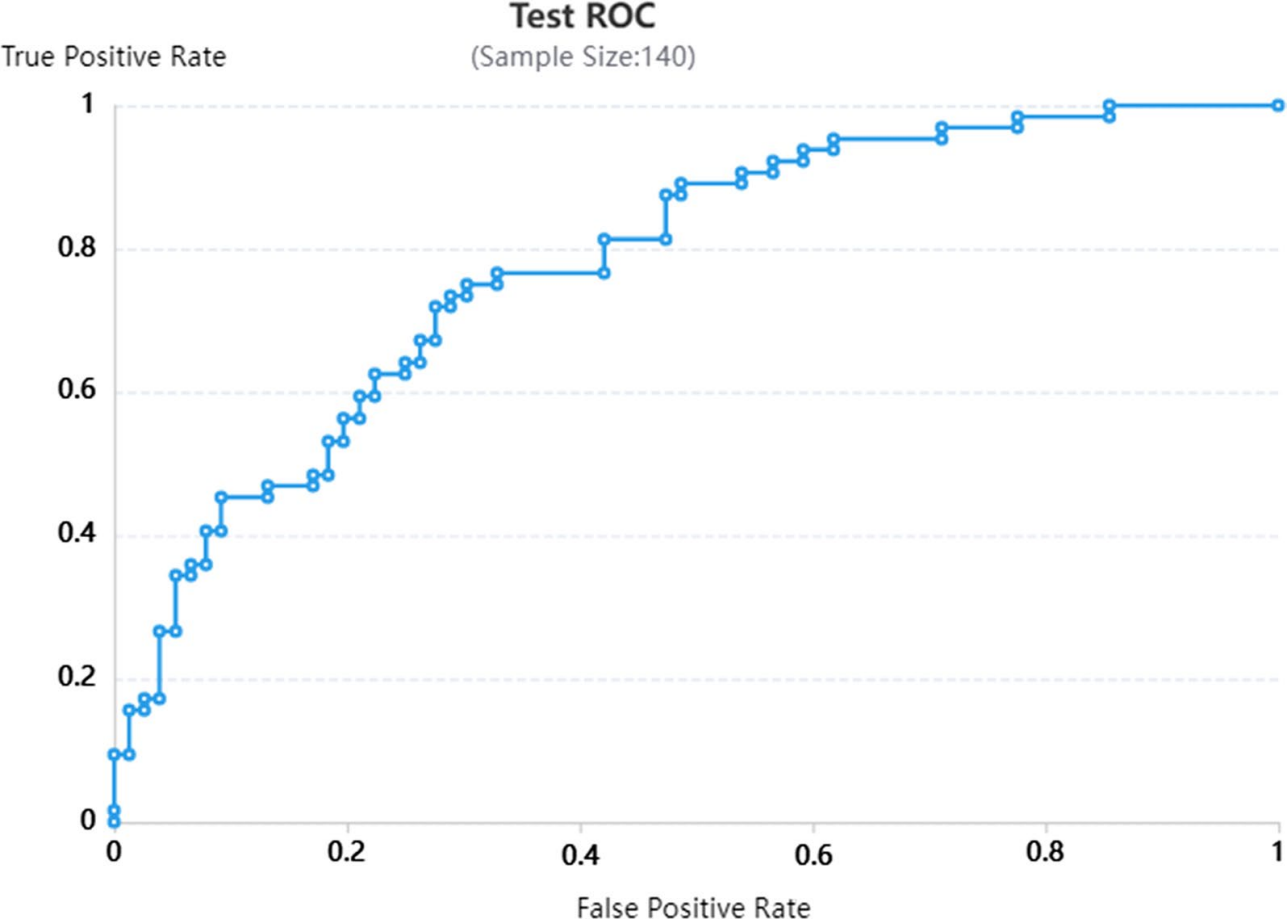
Testing group with the LR radiomics model

Among the enrolled patients, 52 patients with a single lesion and 149 patients with multiple lesions, including them, 73 patients were collected with 5 lesions and 76 patients were collected with 2–4 lesions. The total number of consolidation lesions in the entire dataset was 79, which was difficult to obtain a reliable result when calculated consolidation lesions independently. Therefore, all lesions (699 lesions), nodular lesions (335 lesions) and cavity lesions (285 lesions) were included in the preprocessing and feature screening components, resulting in the identification of 8 important features (all lesions group), as shown in Fig. 4. The eliminated features are shown in Additional file 1: 3. Figure 5 shows the ‘heatmap’ of the selected radiomics features, and remarkable differences in clustering were found between the two-class labels. Subsequently, the features were processed with SVM and LR components to obtain 6 radiomic models for the 3 groups of lesions. The ROC curves are shown in Figs. 6 and 7* (all lesions)* and Additional file 1: 4 and *5 (nodular lesions and cavity lesions)*, and the calibration plot of the LR model in the training and test groups is shown in Additional file 1: 6.

Radiologist-1 and radiologist-2 determined the diagnosis of all lesions, nodular lesions and cavity lesions. The diagnostic efficacy was analyzed and compared with that of the established radiomic models, and the results are shown in *Table **2**.* The AUCs of radiologist-1 for all lesions and cavity lesions alone were 0.77 and 0.74, respectively, which were lower than those of the SVM model (0.79, 0.77) and LR model (0.78, 0.78). The diagnostic efficacy for nodular lesions was between that of the two models (radiologist-1: 0.76, SVM: 0.74, LR: 0.78). The diagnostic efficacy of radiologist-2 was lower than that of the two models (all lesions: 0.63, cavity lesions: 0.67, nodular lesions: 0.65). The diagnostic efficacy of the LR model was relatively stable across the 3 groups (0.78, 0.78, 0.78) and higher in the cavity lesion group and nodular lesion group than in the SVM model group (0.77, 0.74).

**Table 2:** Discriminative performance of the Radiomic model

		Training Cohort			Testing Cohort		
		Sensitivity	Specificity	AUC (95%CI)	Sensitivity	Specificity	AUC (95%CI)
All Lesions	LR	0.792	0.728	0.84 (0.81, 0.87)	0.765	0.667	0.78 (0.70, 0.86)
	SVM	0.842	0.760	0.88 (0.86, 0.91)	0.763	0.719	0.79 (0.71, 0.87)
Cavitation	LR	0.712	0.893	0.87 (0.82, 0.91)	0.681	0.825	0.78 (0.68, 0.89)
	SVM	0.804	0.805	0.88 (0.84, 0.92)	0.722	0.771	0.77 (0.66, 0.88)
Nodule	LR	0.836	0.887	0.93 (0.89, 0.96)	0.815	0.667	0.78 (0.67, 0.88)
	SVM	0.831	0.821	0.91 (0.87, 0.95)	0.850	0.615	0.74 (0.63, 0.85)

**Fig. 8:**
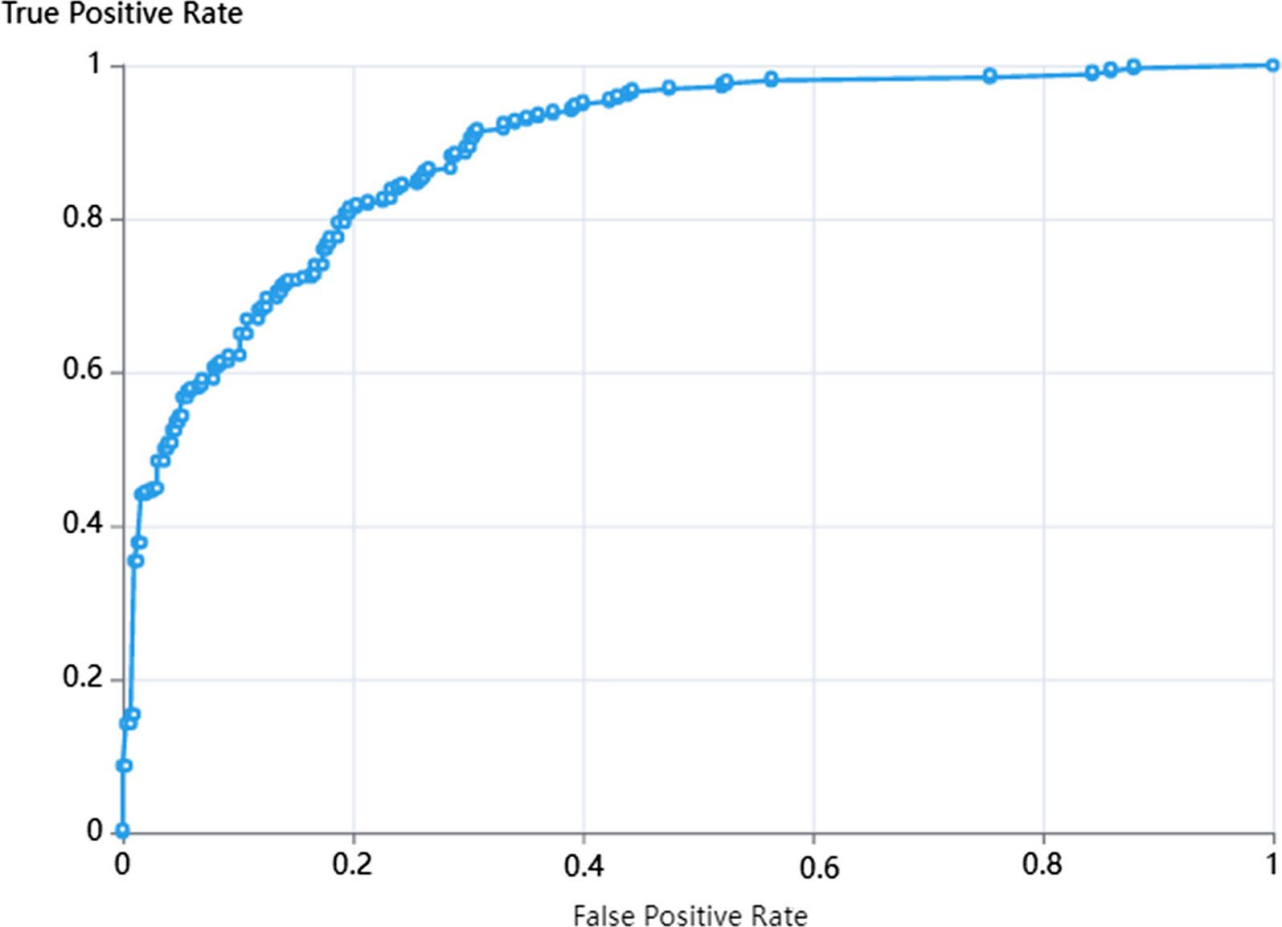
ROC curves for the combination of radiologists and the radiomic model for the diagnosis of all lesions

Finally, radiologist-3 determined the diagnosis of all lesions, nodular lesions and cavity lesions. After the data were incorporated into the input level of the LR model for comprehensive calculation on the platform, the diagnostic accuracy was determined. The AUCs were 0.88, 0.84, and 0.9 for all lesions, nodular lesions and cavity lesions, respectively, which were higher than those of 3 radiologists' empirical diagnosis only as shown in Table 3 and Fig. 8* (all lesions)*.

**Table 3:** Performance of radiologists' empirical diagnostic and combination with radiomic model

	Radiologists' diagnosis						Combine with LR model		
	Radiologist-1			Radiologist-2			Radiologist-3		
	Sensitivity	Specificity	AUC (95%CI)	Sensitivity	Specificity	AUC (95%CI)	Sensitivity	Specificity	AUC (95%CI)
All Lesions	0.750	0.789	0.77 (0.61, 0.93)	0.687	0.579	0.63 (0.55, 0.71)	0.818	0.788	0.88 (0.85, 0.90)
Cavitation	0.732	0.652	0.74 (0.65, 0.82)	0.656	0.660	0.67 (0.58, 0.76)	0.731	0.879	0.84 (0.79, 0.89)
Nodule	0.736	0.661	0.76 (0.66, 0.81)	0.624	0.668	0.65 (0.56, 0.76)	0.755	0.876	0.9 (0.86, 0.94)

## Discussion

At present, studies are being conducted to explore the imaging manifestations of pulmonary Cryptococcus infection in immunodeficient patients, immunocompromised patients and immunonormal patients [7, 11]. For AIDS patients, consolidation and diffuse lesions were generally considered to be the most common imaging findings in earlier studies [9]. Recent studies have suggested that nodules with or without cavitation are the primary manifestation [10]. In our study, we subdivided cryptococcus based on the presence of three characteristics: solid nodules, cavitation and consolidation. The number of patients presenting with cavity lesions (40.78%, 285/699) and solid nodules (47.92%, 335/699) was higher than the number of patients presenting with consolidation (11.30%, 79/699). This may be because, after cryptococcal infection in the lung, macrophages phagocytose spores, resulting in the aggregation of tissue cells, fibroblasts and lymphocytes, which induces delayed hypersensitivity reactions and the formation of granulomatous nodules. CT was performed and revealed the presence of solid nodules and masses. When there is a small amount of focal necrosis in the interior of the lesion and the necrotic material is cleared through the bronchus, various forms of cavity lesions appear. Pathologically, consolidation lesions are mainly composed of a mixture of mycelium and mucinous connective tissue. This pathological change is also the cause or partly the cause of other invasive fungal infections [12–15].

Patients with nonfungal infections or neoplastic lesions were also included in this study, including those with TB, NTM, lymphoma, Kaposi's sarcoma and lung cancer. Tuberculosis is an important cause of high mortality in HIV-positive patients, and 45% are diagnosed through imaging. AIDS patients with TB are prone to both typical imaging manifestations (cavitation, nodules, and lung consolidation) and atypical manifestations such as reversed halo sign (RHS), emphysema or honeycomb changes of interstitial pneumonia [16, 17]. With regard to NTM infection, cavitary and bronchodilated forms are considered to be the dominant imaging manifestations in patients with good immune function, but diffuse lesions and solid nodules are significantly increased in HIV-infected patients [18]. To the best of our knowledge, patients with HIV infection are at a higher risk of developing malignancies associated with these factors, such as immunosuppression, viral coinfection and high-risk lifestyle choices [19]. A subset of HIV-related malignancies are considered to be AIDS-defining, as their presence can be used to confirm the diagnosis of AIDS in an HIV-infected patient, including Kaposi sarcoma, non-Hodgkin lymphoma, and invasive cervical carcinoma. In addition, non-AIDS-defining malignancies such as HL and lung cancer occur at a higher rate in AIDS patients than in non-HIV-infected patients [20, 21]. Although the CT findings of these nonfungal diseases have their own characteristics, there is some overlap in the morphology of nodules, cavitation and consolidation, making identification difficult.

In the present study, the data preprocessing component was used to stretch, standardize or normalize the original feature vectors. A ‘standard scaler’ was selected as the preprocessing component, which projects all data onto a unit ball for normalization. To eliminate the many redundant features found in the original data, feature selection components were utilized. ‘Select K percentile’– ‘recursive feature elimination’—‘select from model’ were carried out sequentially. Three calculation criteria were set up in the ‘select K percentile’ component, namely, chi2, f_classif and mutual_info_classif, and after screening, the number of features was reduced from 1671 to 267. Regarding ‘recursive feature elimination’, by removing the features with the lowest importance from each round of training, the classifier was trained repeatedly until the classification performance began to decline or reached the minimum number of features. After this step, the number of features decreased to 107. With regard to the established model, the ‘select from model’ component is used to calculate the importance score of the input features and to filter out the features below the threshold. Finally, 8 features with the highest diagnostic value were obtained. In the processor component, SVM and LR were selected to build two different models to compare the diagnostic performance. SVM is a dichotomous model that seeks decision surfaces by maximizing classification intervals in feature spaces. It is very efficient in high-dimensional space and is suitable for small-sample learning tasks. It uses a subset of the training set in the decision function, so it is also very efficient in the use of storage. For visualization, a ‘heatmap’ was used to display the difference in feature distribution in different categories so that the values of each one-dimensional feature of the sample can be reflected in the thermogram in color.

In the study, consistency tests of radiologist-1 and radiologist-2 for border recognition, density, and morphology of lung lesions showed that the interclass correlation coefficient value was 0.819–0.862, reflecting the reproducibility and applicability of image processing in the present research. After radiologist-1 and radiologist-2 made the final diagnosis of all lesions based on CT characteristics, the diagnostic performance was calculated and analyzed. The diagnostic efficacy of the LR model was higher than that of the SVM in the group of cavity lesions and nodular lesions. The diagnostic efficacy of both the SVM model and LR model for AIDS complicated by pulmonary cryptococcus infection was similar to that of Senior radiologist for pulmonary infectious diseases and higher than that of the primary radiologist. Finally, the data of radiologist-3 were incorporated into the input level of the LR model for comprehensive calculation on the platform. The AUCs for all lesions, nodular lesions and cavity lesions were higher than those of both radiologists’ empirical diagnosis and the radiomic model only.

Despite the promising results, several limitations should be discussed. Firstly, this was a single-center retrospective study that included 203 cases with 699 lesions totally, even though we've included more patients than most studies of AIDS with pulmonary cryptococcosis, multi-institutional data was still needed to be incorporated into the model to evaluate the generalisation of the results. Secondly, the radiomics features in this study were derived from ‘Darwin Platform’ from an AI technology incorporating PyRadiomics library to extract abundant radiomics features, the discrepancy between this software and other open-source libraries, like Imaging Biomarker Explorer (IBEX), for radiomics feature extraction was still needed to be compared to ensure the reproducibility of study. Finally, the performance of the model was only compared and evaluated with three radiologists of different seniority to interpret the lesions of AIDS complicated with pulmonary cryptococcosis using a limited dataset, which may not reflect actual radiologist ability.

## Conclusions

The CT-based radiomic LR model of AIDS-associated pulmonary cryptococcosis exhibits good diagnostic performance, which was similar to that of senior radiologists and higher than that of the primary radiologist. With the help of a radiomic model, radiologists can achieve improved diagnostic accuracy compared to that when only an empirical diagnosis is used.

## Supplementary Information

**Additional file 1:** Extract parameter details and the results of each step of feature extraction.

## Data Availability

The datasets generated or analyzed during the current study are available from the corresponding author on reasonable request.

## Abbreviations

AIDS: Acquired immune deficiency syndrome
ROC: Receiver operator characteristic curve
SVM: Support vector machine
LR: Logistic regression
AUC: Area under the curve
IPFI: Invasive pulmonary fungal infections
HIV: Human immunodeficiency virus
ROI: Region of interest
CD4: Cluster of differentiation 4
CD8: Cluster of differentiation 8
NTM: Non-tuberculous mycobacteria
DICOM: Digital imaging and communications in medicine
GLDM: Gray-level dependence matrix
GLSZM: Gray-level size zone matrix
GLCM: Gray-level cooccurrence matrices
GLRLM: Gray-level run-length matrices
NGTDM: Neighboring gray-tone difference matrix
CI: Confidence interval
IQR: Interquartile range
ICC: Intraclass correlation coefficient
TB: Tuberculous mycobacteria
AIC: Akaike information criterion
