# Supplementary material for: Establishment and evaluation of a CT-based radiomic model for AIDS-associated pulmonary cryptococcosis
Source: BMC Med Imaging. 2022 Oct 29;22:185. doi: 10.1186/s12880-022-00910-6 (PMC9617378; doi:10.1186/s12880-022-00910-6)
Supplement: Supplementary file 1 — Additional file 1: Extract parameter details and the results of each step of feature extraction. [file 12880_2022_910_MOESM1_ESM.docx]

**1. Extraction parameter details**

A total of 1781 radiomic features were extracted automatically, the original feature classes contain shape, first-order and texture features. First-order features refer to the distribution voxel intensities through ommonly used and basic metrics, such as mean, range, variance and kurtosis. Texture features can describe the describe the heterogeneity of the lesions, which include gray-level cooccurrence matrix (GLCM), gray-level run length matrix (GLRLM), gray-level size zone matrix (GLSZM), neighboring gray-tone difference matrix (NGTDM), and gray-level dependence matrix (GLDM). The original images were normalized with the window width and window level of 1600HU and -600HU, and then rescaled to 0-2048 for discretization. A fixed bin width of 25 was used in gray-level discretization. Eight types of transformed images which derived from the original images using different filters were further used, including Laplacian of Gaussian (LoG), wavelet, square, square root, logarithm, exponential, gradient, and local binary pattern (LBP). Features mention above can also be extracted from the derived images (except shape features). Missing values were filled with previous value, data preprocessing method-standard scaler were applied to standardize features by removing the mean and scaling to unit variance. The optimal feature filter (sample variance F value) were used to evaluate the linear correlation between each feature and category label, and 321 most relevant features were selected from 1781 features. Some machine learning classifiers themselves can evaluate feature importance and find the best feature combination through multiple iterative calculations, so recursive feature elimination (RFE) based on support vector machine (SVM) was further applied to obtain the optimal predictive feature combination step by step based on accuracy, 20 least important features below threshold were eliminated each literation, at least 7 features retained. The performance of the models was further tested in independent validation set, using the same threshold determined in the training set, with the threshold established by maximizing the Youden index. Area under the ROC curve and 95% confidence interval were calculated. The diagnostic performance of radiomics models was compared by Delong’s test. A detailed description of the imaging omics components is available on the PDF file: arXiv:2009.00908v1.
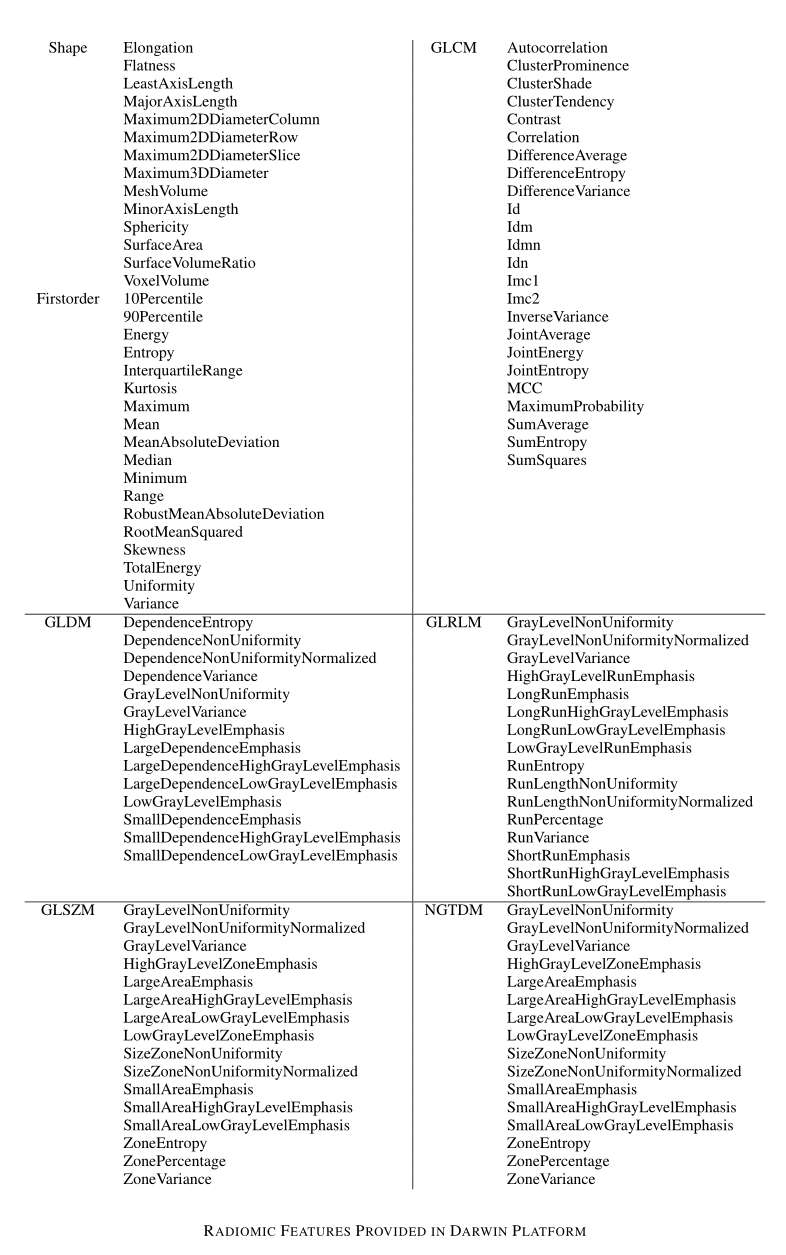


**2. ‘Recursive feature elimination’ components of LR model.
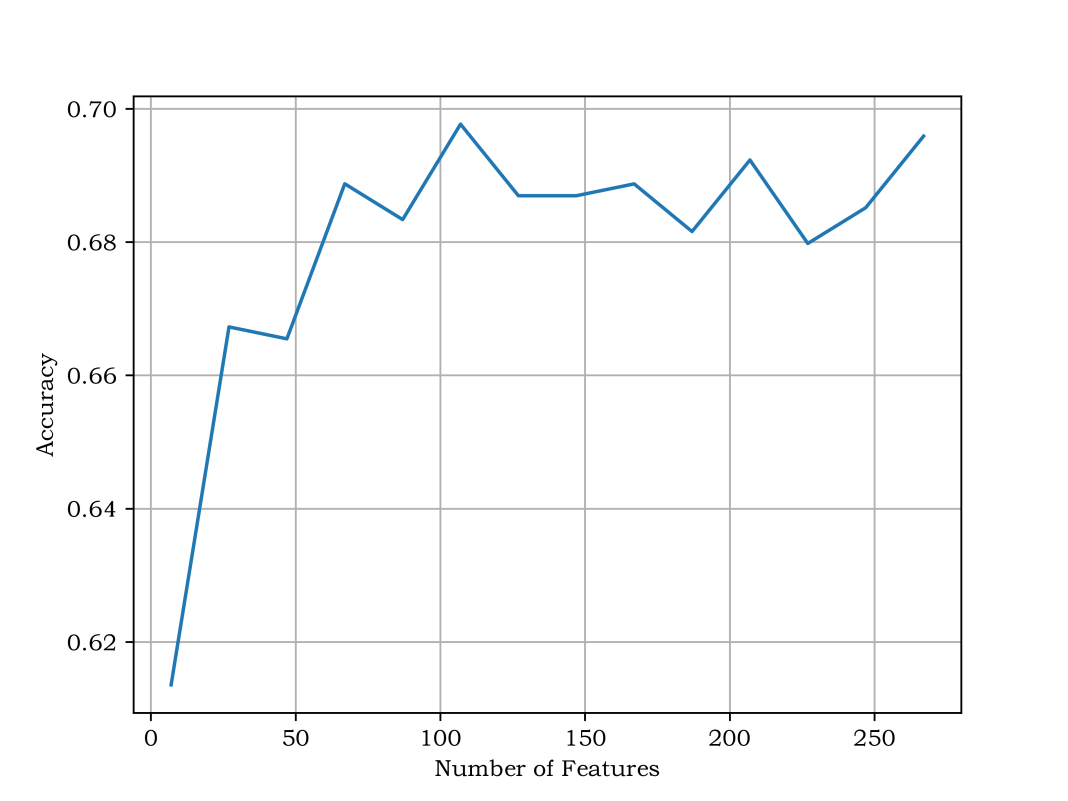
**

**3.** **Eliminated features from ‘Select K percentile’ components
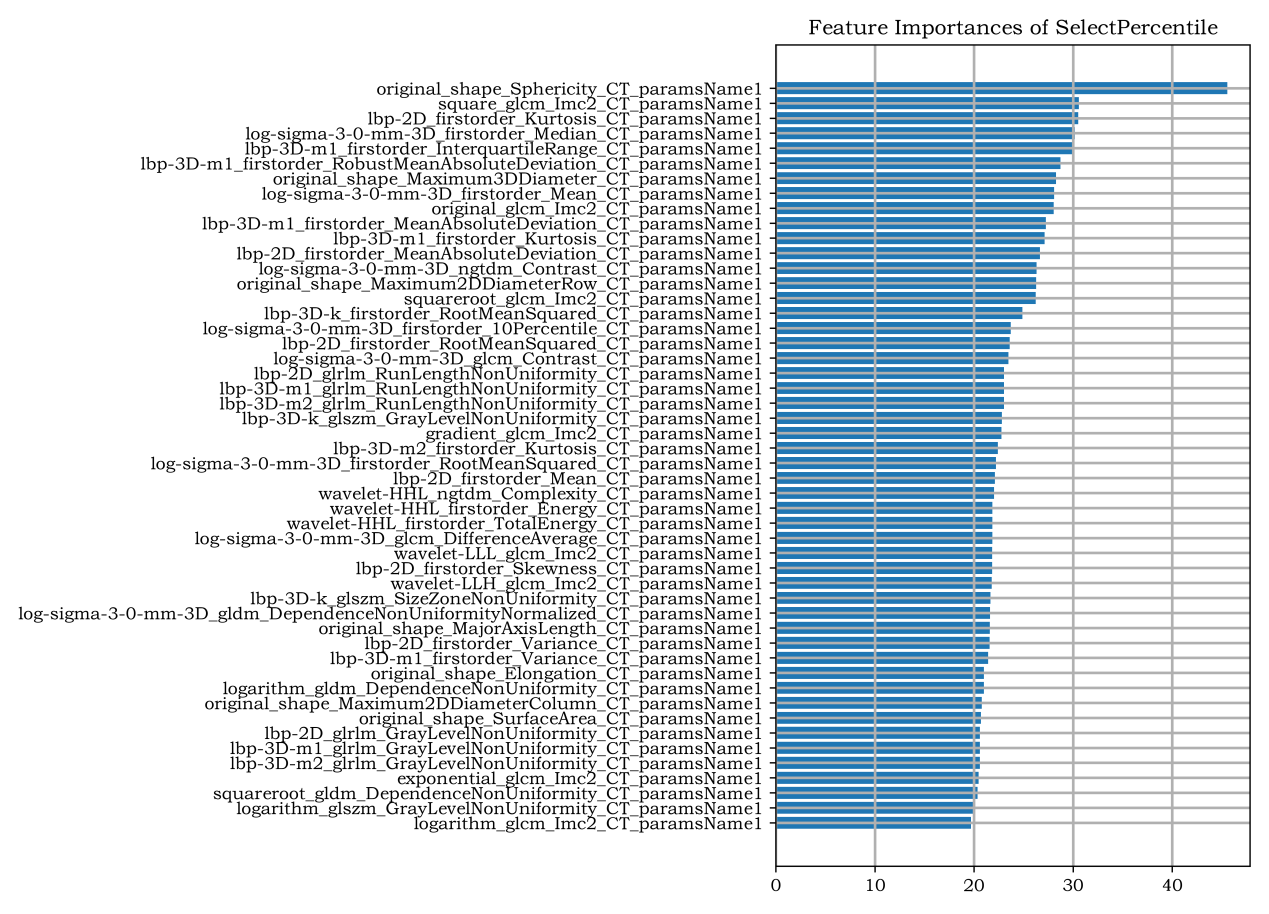
**

**4. Training group and test group of cavitation**


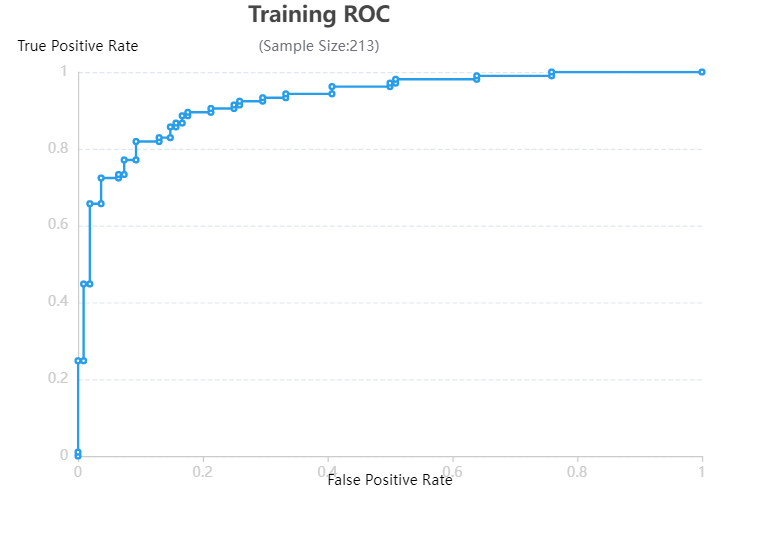


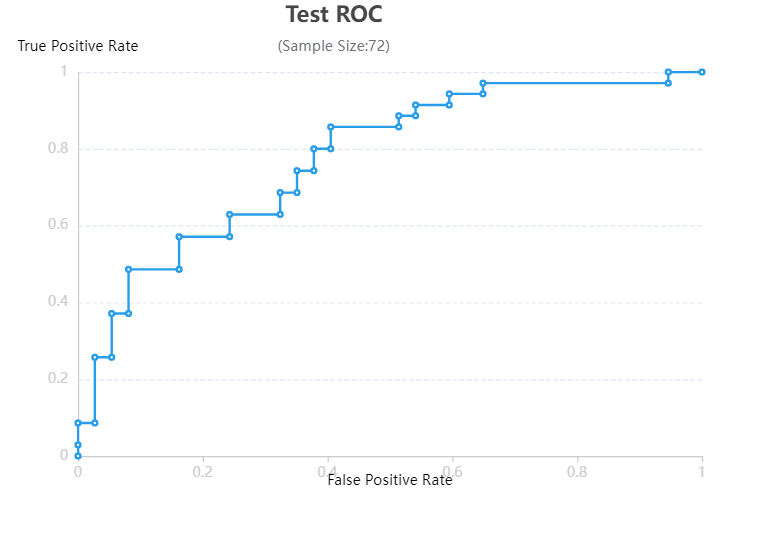


**5. Training group and test group of nodule.**


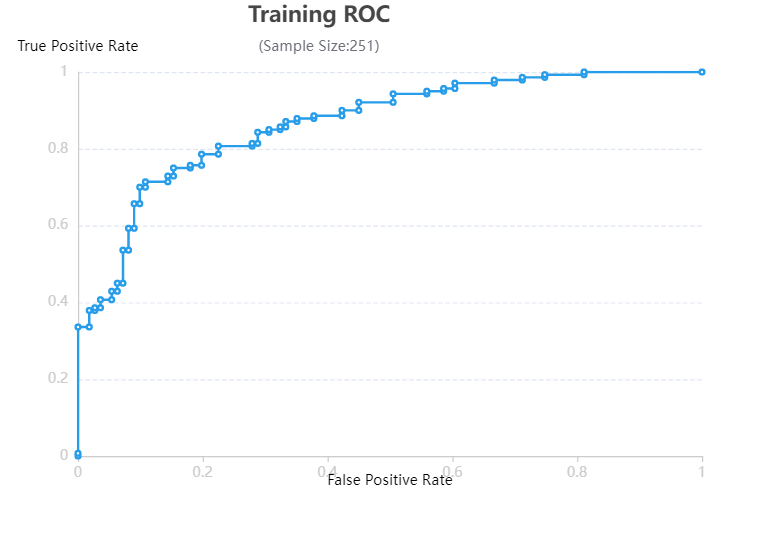


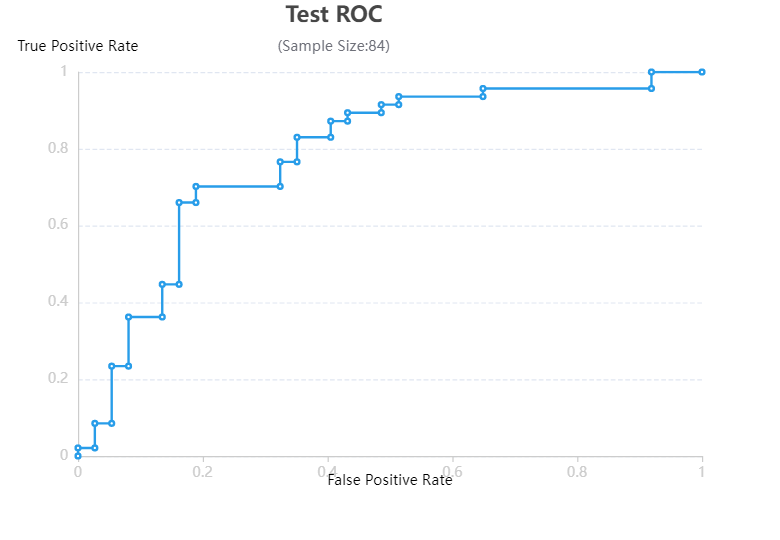


**6. The calibration plot of LR model**

**
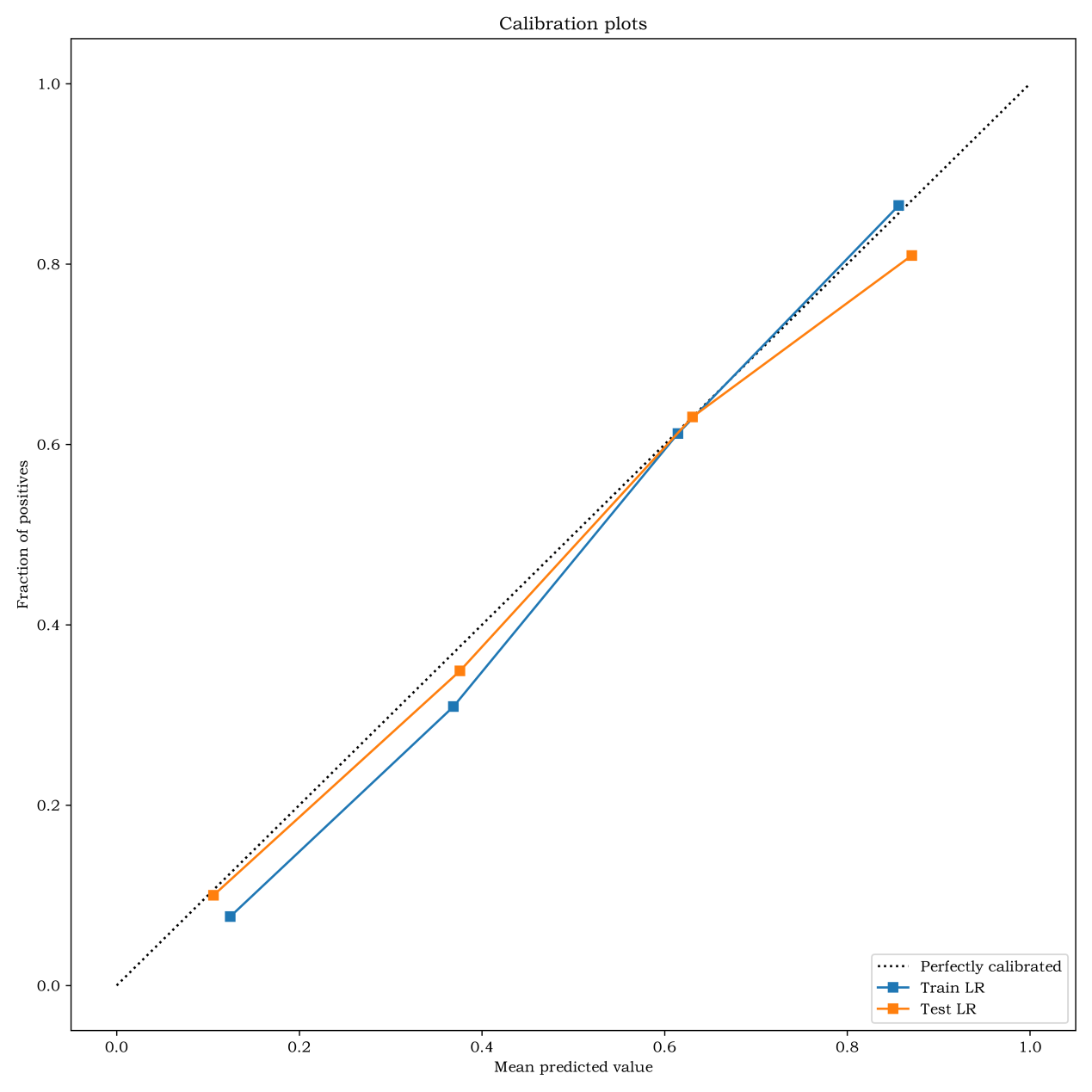
**
